# Life stage shapes condition-dependent foraging under predation risk in an electric fish

**DOI:** 10.1098/rsos.260914

**Published:** 2026-10-21

**Authors:** Lok Poon, William G. R. Crampton

**Affiliations:** Department of Biology, University of Central Florida, Orlando, FL 32816, USA

**Keywords:** antipredator behaviour, asset protection principle, body condition, electrolocation, life-history trade-offs, lunar illumination, risk-sensitive foraging, sensory ecology, state-dependent behaviour, terminal investment

## Abstract

Low body condition can promote risky foraging, but whether condition dependence differs across life stages remains unclear. Using field and laboratory studies, we tested whether residual body condition (RBC) predicted foraging under moonlight-mediated predation risk in the Amazonian sand knifefish, *Gymnorhamphichthys rondoni*. This species has a 2-year lifespan and a single late second-year breeding season. Because adults have less time than juveniles to recover condition before breeding, we predicted stronger condition dependence in adults. Among wild fish pooled across life stages, stomach fullness increased overnight on dark (lower-risk) but not bright moonlit (higher-risk) nights, when most stomachs were empty and non-empty stomachs occurred mainly in low-RBC fish. In experiments under laboratory-recreated moonlight, lower-RBC adults were active longer than higher-RBC adults on full-moon nights, as quantified by electric organ discharge rate. RBC did not predict adult activity on new-moon nights or juvenile activity on full- or new-moon nights. A full-moon interaction model showed a significantly stronger RBC–activity relationship in adults than in juveniles. In light-ramping trials, lower-RBC adults remained active at higher illuminance before hiding, whereas most juveniles did not hide. Our results are consistent with the asset-protection prediction of stronger condition-dependent risk-taking in adults facing late-life constraints than in juveniles.

## 1. Introduction

Behavioural decisions depend not only on environmental factors but also on an individual's internal state, such as energetic reserves or physiological condition [1]. Understanding how internal state shapes decisions involving substantial risk remains a central problem in behavioural ecology [2–4]. Foraging exemplifies this challenge: acquiring food supports growth, survival, and reproduction, but often increases exposure to predators. Because foraging opportunities vary in predation risk, individuals must balance energetic gain against safety [5]. Although foraging under safer contexts is generally favoured, individuals in certain states may nonetheless accept elevated predation risk to meet energetic demands [6,7]. How such condition-dependent risk-taking is expressed at different life stages remains poorly understood.

Body condition is a widely used proxy for energetic reserves, and higher condition is often associated with increased survival [8] and reproductive success [9]. Under the asset protection principle, individuals in low condition should accept greater predation risk to recover energetic deficits, whereas those in high condition should restrict foraging to safer contexts to protect reproductive potential [10]. Consistent with this framework, foraging effort under predation risk is often predicted by nutritional state or body condition [11–13]. For example, low-condition scorpions (*Buthus occitanus*) forage more than high-condition individuals during predator-prone moonlit periods [13], while underfed goldfish (*Carassius auratus*) take greater foraging risks than well-fed individuals in the presence of a little egret (*Egretta garzetta*) [11].

Life stage can further modify risk-taking because juveniles and adults differ in how they allocate effort between safety, growth and reproduction [14–16]. Adults are often expected to be relatively risk-averse to protect reproductive assets, whereas juveniles may accept greater risk to promote growth [10,17]. Consistent with this expectation, a meta-analysis reported that condition-dependent risk-taking is commonly stronger in juveniles than in adults [12]. However, the asset protection principle does not intrinsically predict stronger condition dependence in either life stage. Rather, the relative strength of condition dependence across life stages should depend on life stage-specific fitness pay-offs shaped by residual reproductive value and time constraints. Under some life histories, particularly those involving strong late-life time constraints, condition dependence may therefore be stronger in adults than in juveniles [4,18,19]. Although condition-dependent risk-taking has been widely studied, relatively little is known about how body condition shapes risk-taking at different life stages [12]. Direct tests of how poor body condition, rather than acute hunger, influences foraging under predation risk at different life stages remain rare. The closest relevant evidence comes from 13-lined ground squirrels (*Ictidomys tridecemlineatus*), in which nutritional need helps explain why juveniles are less vigilant during foraging than adults [20], and North American porcupines (*Erethizon dorsatum*), in which winter nutritional stress affects the use of risky foraging habitats differently among age classes [21]. Addressing this gap requires a study system with a well-characterized life history and ecologically grounded measures of body condition, foraging behaviour and predation risk.

We investigated how body condition affects foraging under predation risk in the sand knifefish, *Gymnorhamphichthys rondoni* (Teleostei, Gymnotiformes), a small (< 25 cm), weakly electric fish inhabiting shallow, clear-water Amazonian streams [22–26]. Individuals remain buried during the day and emerge after sunset to forage, using active electrolocation and passive electroreception to locate benthic invertebrates. They forage throughout dark periods but strongly suppress emerged activity and hide in the sand under moonlight [22,26], forgoing approximately one-quarter of the available nocturnal foraging time over a lunar cycle [22]. This response is highly sensitive: activity is reduced by approximately 80% at illuminances as low as 4 × 10^−4^ lx [22,26]. It is also temporally precise, tracking variation in ground-level illumination arising from lunar phase, daily shifts in moonrise and moonset, the moon's ascent and descent, canopy-horizon obstruction and cloud cover [22,26].

Several lines of evidence support interpreting this response as antipredator behaviour rather than as a generalized physiological suppression of activity by light. In a comparative field study at a Colombian rainforest stream, four co-occurring electric fish species with similar diets and foraging habitats differed markedly in their moonlight responses, which covaried with body mass-adjusted eye size. The two smallest-eyed species, including *G. rondoni*, exhibited strong moonlight avoidance, whereas the largest-eyed species remained active under moonlight [22]. At the same stream, visually oriented nocturnal piscivores, including nocturnal herons, water opossums (*Chironectes minimus*) and trahira fish (*Hoplias malabaricus*), were abundant [22]. Controlled laboratory experiments also demonstrated rapid, reversible transitions between emerged activity and hiding when simulated moonlight was removed or restored while all other environmental variables were held constant [26]. Together, these findings support the use of moonlight as a finely graded and ecologically relevant index of visual predation risk in *G. rondoni*.

Sand knifefish provide an opportunity to examine condition-dependent foraging at different life stages under a natural gradient of light-associated predation risk. Many previous studies have relied on feeding treatments as coarse proxies for energetic state or on predation-risk manipulations that are difficult to relate to natural risk gradients [27–29]. In sand knifefish, body condition can be quantified relative to a wild population, and nocturnal moonlight provides a finely graded index of visual predation risk [22]. In addition, the pulse rate of the continuously emitted electric organ discharge (EOD)—the fish's active electrosensory sampling rate [30]—increases from rest while buried (approx. 10–40 Hz) to active swimming (approx. 70–100 Hz), with an abrupt step increase at emergence from the sand [24,26]. This stereotyped shift enables quantification of buried and emerged activity states at high temporal resolution.

Sand knifefish also exhibit a life history that generates contrasting predictions across life stages. They live up to 2 years in the wild, with a non-breeding first year and reproduction concentrated in the second year during a protracted rainy-season breeding period comprising multiple spawning events [25,31]. Second-year adults are not detected after the rainy-season breeding period, consistent with post-reproductive mortality [25,31,32] and suggesting that reproduction is effectively confined to a single terminal season. In non-breeding fish, foraging constitutes the principal sustained nocturnal activity [22]. Our laboratory experiments included juveniles (first-year fish below the minimum maturation length) and adults (late second-year fish above this threshold but not yet capable of spawning).

Based on this life history, we predict that adult sand knifefish should show strong condition-dependent modulation of foraging under predation risk, with low-condition individuals accepting greater risk than high-condition individuals (figure 1). Adults approaching a terminal breeding season have limited time to improve energetic state. Low body condition, which can constrain reproductive output, may therefore favour acceptance of predation risk to recover energetic deficits [4,18]. By contrast, adults in high condition should restrict foraging to safer contexts to protect reproductive potential.

**Figure 1.**
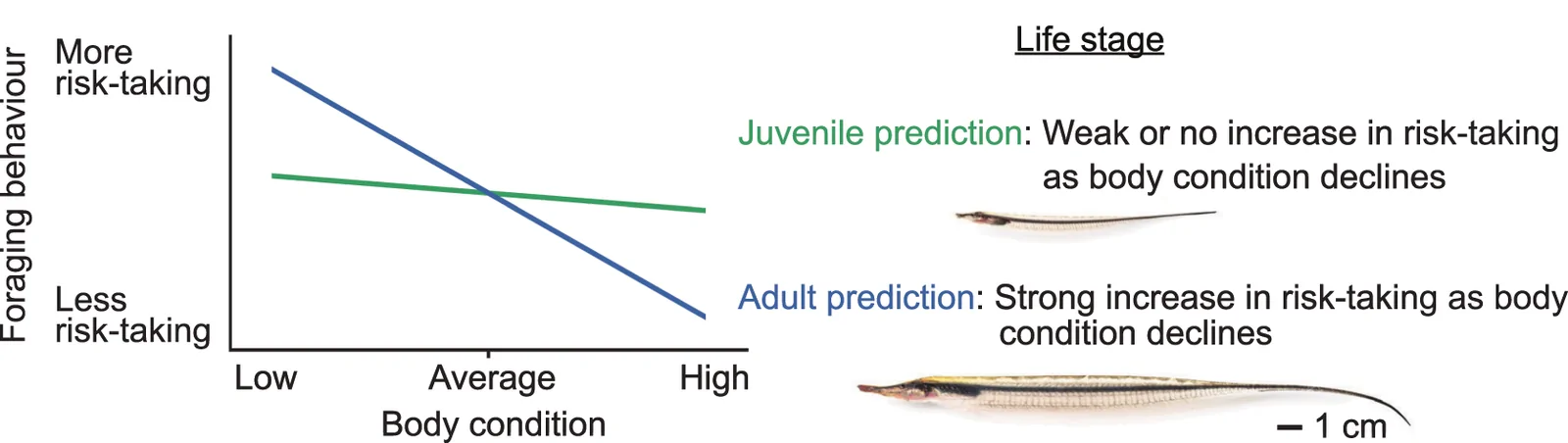
Conceptual predictions for condition-dependent risk-taking in adult and juvenile sand knifefish. Schematic of predicted relationships between body condition and risk-taking when foraging under predation risk. Life stage differences in overall risk-taking (intercepts) are not central to these predictions. The predicted contrast concerns the adult and juvenile slopes, which were compared directly in the secondary full-moon residual body condition (RBC) × life stage interaction analysis (model 7A).

In juveniles, we predict weak or absent condition-dependent modulation of foraging under predation risk (figure 1). Foraging decisions in juveniles are expected to reflect a trade-off between growth and safety, with the balance shaped by baseline juvenile mortality [18,33,34]. When juvenile mortality is high, selection for rapid growth can favour foraging under greater predation risk [35]. When mortality is low, the associated survival benefit of accelerated growth is reduced, and selection for condition-dependent risk-taking is correspondingly weaker [36]. Mark–recapture studies report high juvenile recapture rates in sand knifefish, suggesting low juvenile mortality [31]; this is consistent with their sand-burrowing daytime behaviour. Given this low mortality (and because they live year-round in systems that do not impose pronounced seasonal energetic constraints [37,38]), juvenile sand knifefish are not expected to show strong condition-dependent modulation of foraging under predation risk [39–41].

To test these predictions, we combined field sampling of wild sand knifefish with controlled laboratory experiments. In wild fish, we tested at the population level whether the relationship between body condition and recent feeding, indexed by stomach fullness, differed between dark (lower-risk) and bright moonlit (higher-risk) nights. In the laboratory, we maintained separate groups of juvenile and adult fish under recreated lunar illumination cycles and manipulated food intake to generate residual body condition (RBC) values spanning the range observed in the wild. Using continuous recordings of EOD rate to infer activity, we quantified individual risk-taking with two assays: (i) active minutes on bright full-moon versus dark new-moon nights and (ii) the illuminance at which fish hid during repeated light-ramping trials. Stage-specific models provided our primary tests of RBC–activity relationships within each life stage and moon phase. For each moon phase, we also fitted a secondary active-minute model including both life stages and an RBC × life stage interaction to compare the adult and juvenile RBC–activity slopes directly. Together, these approaches allowed us to test for condition-dependent foraging under predation risk at the population level in the wild and to evaluate both stage-specific patterns and adult–juvenile differences in a controlled laboratory setting.

## 2. Materials and methods

### 2.1. Wild fish: sampling and electric organ discharge recordings

A total of 292 sand knifefish (figure 2a) were collected from rainforest streams near Jenaro Herrera, Peru (04°54′ S, 073°40′ W [42]) during the mid-dry season (July–August 2018), when reproductive activity is minimal [32]. Fish were sampled on 16 nights between 19.00 (approx. 20 minutes after the end of nautical twilight) and 00.00. Ground-level moonlight illuminance at the time and location of capture was estimated, rather than measured *in situ*, using *MoonShineR*, an R package that predicts moonlight ground illuminance at user-defined intervals for a specified geographic location and time range using astronomical values and equations [43]. Predictions assumed an open, cloudless sky without attenuation by canopy. Nights were classified as ‘bright’ (full moon and the preceding three nights; *n* = 59 fish) when predicted illuminance exceeded 0.03 lx throughout the 19.00–00.00 sampling period, or as ‘dark’ (*n* = 233 fish) when predicted illuminance remained <0.01 lx, typically 0 lx, throughout the sampling period.

**Figure 2.**
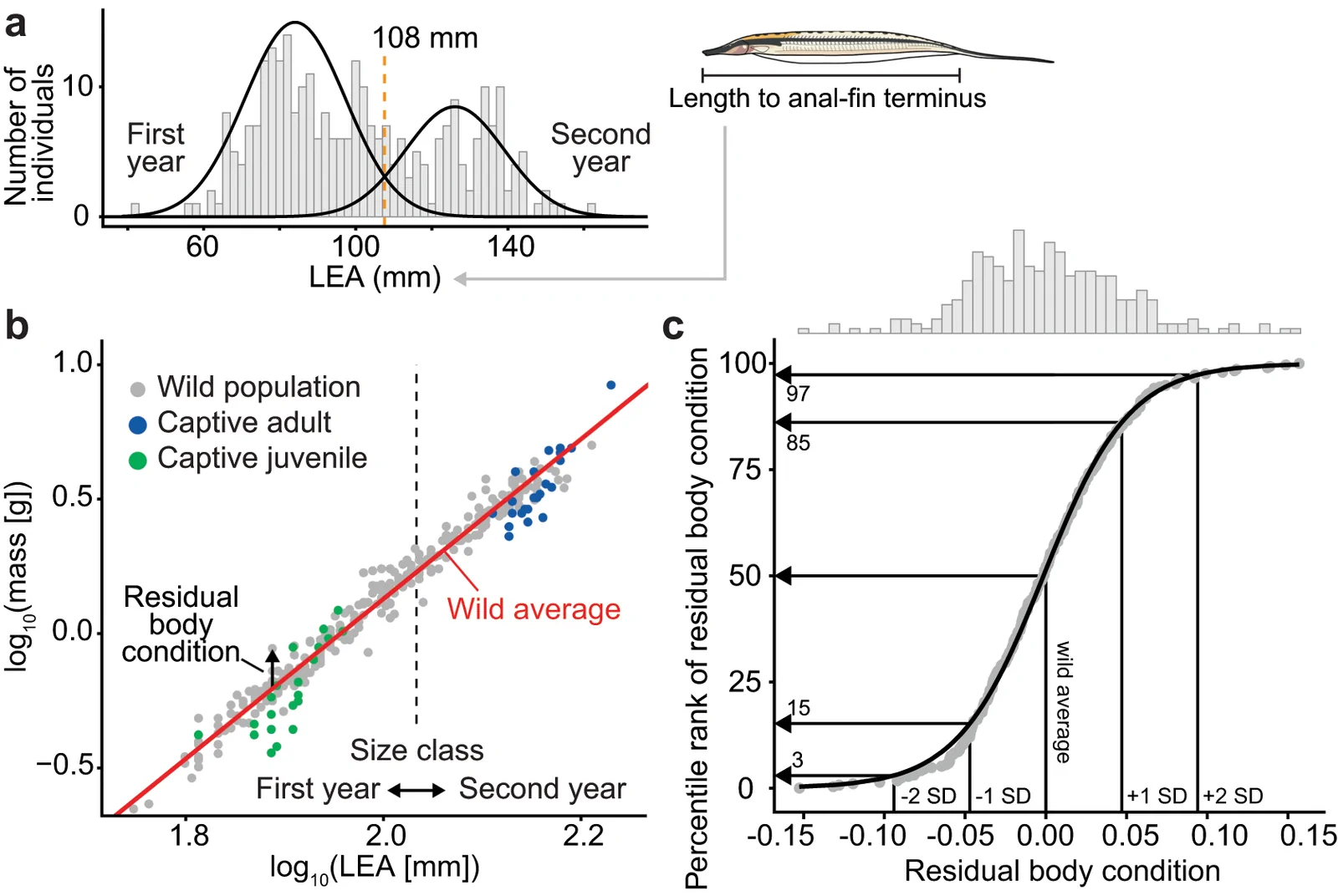
Length and body condition of wild and captive sand knifefish. (a) Frequency distribution of length to anal-fin terminus (LEA) in the wild population (bin width 2 mm; *n* = 292), showing the overlapping first- and second-year length distributions estimated by mixture modelling, with a crossover at 108 mm. (b) Allometric relationship between log_10_(mass) and log_10_(LEA) for wild fish (grey, *n* = 292) and captive fish (adults blue, *n* = 23; juveniles green, *n* = 21) during the experiment's final full moon (5th full moon for adults, 2nd full moon for juveniles; figure 3a–b). Red line: wild-population standardized major axis (SMA) regression; RBC is the vertical deviation of an individual (wild or captive) from this line. (c) Logistic relationship between RBC and percentile rank within the wild population. RBC = 0 corresponds to the wild average, which approximately matches the 50th percentile. Histogram (top): wild RBC distribution (bin width 0.0063). Vertical lines: mean, ±1 s.d. and ±2 s.d.

Fish were located by detecting their continuously emitted EODs with an electric fish finder [44] while the collector waded slowly upstream using a dim red LED headlamp. Although EOD rate differs between activity states, EOD amplitude—and therefore detection range—does not vary with EOD rate and remains similar between active and buried fish of comparable size. Fish in both states were therefore readily detectable. Active fish also swam slowly (typically 0.03–0.08 m s^−1^) and never fled upon approach, minimizing the likelihood of activity-dependent sampling bias.

Once located, each individual's EOD was recorded for 60 seconds at close range using orthogonally mounted electrode pairs on a hand-held rod. Recordings began at approximately 0.5 m from the fish, and EOD rate showed no apparent change during approach or recording. Each electrode pair was connected to a BMA-200 bioamplifier (CWE, Ardmore, PA, USA). Amplifier outputs were digitized with a USB-6216 analogue-to-digital converter (NI, Austin, TX, USA) at 50 kHz (16-bit) and acquired using custom LabVIEW software on a waterproof tablet PC mounted on a chest harness. Mean EOD rate was calculated by rectifying and summing the two input channels and applying peak-detection algorithms in a custom LabVIEW program [26]. Fish were captured with a dip net immediately after EOD recording, euthanized by eugenol overdose, fixed in 10% formalin for two–three weeks and preserved in 70% EtOH. From preserved specimens, we measured stomach fullness (§2.2) and body mass and length (§2.3).

### 2.2. Wild fish: stomach fullness

We calculated the quantitative stomach-fullness index (QSF), following Marsac & Potier [45]: QSF = (stomach-content mass / (empty-stomach mass + stomach-content mass)) × 100. Because sand knifefish typically begin foraging shortly after nightfall, QSF at capture provides an index of recent feeding and hence of foraging success during the current night. Capture time was expressed as minutes since the end of nautical twilight. We avoided stomach-fullness indices scaled to total body mass because stomach contents comprised only a small fraction of body mass, compressing ratios towards zero—especially when content mass was low. Scaling stomach-content mass to the combined mass of the stomach and its contents provided greater resolution.

Stomachs were dissected from EtOH-preserved specimens by excising the alimentary tract and cutting the oesophagus and intestine at their junctions with the stomach. Each intact stomach (with contents) was blotted to remove excess preservative and weighed to ±0.001 g using a Sartorius 64-1S microbalance (Sartorius AG, Göttingen, Germany). Contents were then removed, consolidated into a single bolus, blotted and weighed to ±0.001 g on the same balance.

### 2.3. Wild and captive fish: residual body condition

Body condition was quantified as RBC, defined as each individual's residual relative to the wild-fish allometric mass–length relationship (figure 2b). Mass–length residual indices of body condition are widely used as morphometric proxies of energy reserves in fishes [46].

Wild fish body mass and length were measured after two–three weeks of formalin fixation (before EtOH storage). Mass was measured to ±0.01 g using a digital balance. Body length was expressed as snout-to-anal-fin terminus length (LEA) and measured to ±0.5 mm using a ruler. Total length was not used because sand knifefish commonly exhibit damage or regeneration of the caudal filament. We then fitted a standardized major axis (SMA) regression between log_10_(mass) and log_10_(LEA) to establish the wild allometric mass–length relationship (*n* = 292) (figure 2b). Because formalin fixation slightly increased mass relative to live fish, log_10_(mass) was corrected to live equivalents by subtracting a constant of 0.01428, based on a prior comparison of fresh versus fixed specimens; body length was unaffected by fixation.

RBC was calculated for all individuals, wild and captive, as the residual from this wild SMA regression: observed log_10_(mass) minus expected log_10_(mass) for the individual's LEA. For interpretability, body condition was also expressed as (i) percentile rank of RBC within the wild population (50th percentile = median; figure 2c) and (ii) percentage difference between observed and expected mass.

### 2.4. Captive fish: acquisition and husbandry

The captive fish used in laboratory experiments were wild-caught and imported directly to the Crampton Lab from an ornamental fish dealer in Iquitos, Peru. The fish were exported approximately two weeks after capture and approximately two weeks before the start of the approximately three-month acclimation period described in §2.9 below. They were quarantined on arrival for approximately one week and treated prophylactically with formalin, praziquantel and fenbendazole to control bacterial, protistan and helminth infections. Fish were maintained individually for continuous EOD recording throughout the experiment, each housed in a 76 l tank (61 × 30 × 41 cm) containing 4 cm of silica sand. The solitary nature of sand knifefish suggests that individual housing does not affect behaviour [23]. To minimize electrical interference, the experimental room was temperature-controlled (avoiding immersion heaters), and tanks were filtered with air-driven sponge filters. Water temperature (24.5 ± 0.2°C), pH (6.0–7.5) and conductivity (20–40 µS cm⁻¹) were maintained to approximate rainforest stream conditions; nitrogenous waste was monitored daily.

### 2.5. Captive fish: life stage classification

We classified captive individuals into juvenile and adult life stages based on body size and gonad morphology. Captive experiments included 21 juveniles (65–91 mm LEA) and 23 adults (129–170 mm LEA), with no individuals in the intermediate size range (figure 2a,b). Juveniles were defined as first-year individuals below the minimum size at which gonadal maturation occurs (105 mm LEA [31]). Adults were defined as individuals at or above this threshold (LEA ≥105 mm); all adults were in the upper range of the second-year size class. Sex and gonadal maturation stage (stages 1–4 [32,47]) were determined by visual examination of the gonads through the transparent ventral body wall over the coelom. Adults showed early-stage maturation (stages 1–2), and none reached full sexual maturation during the experiment (stages 3–4). Juveniles could not be sexed.

### 2.6. Captive fish: electric organ discharge recording

Each tank contained four orthogonally arranged electrode pairs to detect EODs regardless of fish orientation (supplementary material, figure S1). Following Poon & Crampton [26], signals were rectified, summed across electrode channels, amplified with custom electronics, digitized with a USB-6363 analogue-to-digital converter (NI, Austin, TX, USA) and recorded as 15-s WAV files at 1-minute intervals using a LabVIEW program. Mean EOD rate for each file was extracted using peak-detection algorithms in the same program used for field recordings (§2.1).

### 2.7. Captive fish: recreation of lunar illumination

Fish were maintained on a 12 : 12 hour light–dark solar photoperiod (185 : 0 lx) using daylight-balanced fluorescent lights controlled by a Lutron Grafik-Eye system, with 30-minute ramps at lights-on and lights-off. The 12-hour light phase approximated natural day length in near-equatorial Amazonia, while the 30-minute ramps approximated the region's rapid dawn and dusk [43]. Moonlight cycles were recreated separately using the *MoonShineP* system, in which a Raspberry Pi computer controlled an individually addressable LED array with a moonlight-like spectrum. Cycle timing followed equatorial open-sky predictions for the experimental dates (2 April–23 November 2021), and illuminance was updated every minute (figure 3a,f). The programmed cycles reproduced an approximately 50-minute night-to-night delay in moonrise and moonset, the increase in peak illuminance towards the full moon and the within-night changes in illuminance associated with lunar ascent and descent [43]. Illuminance on the chamber floor was logged every 15 seconds with an ILT5000 low-light meter (International Light, Peabody, MA, USA) fitted with an SED 100-10/U photosensor, Y4 photopic filter and wide-angle diffuser (detection limit 2 × 10^−4^ lx; accuracy ±2 × 10^−4^ lx). The LED array was calibrated to a maximum full-moon illuminance of 0.062 lx, matching natural stream-level moonlight beneath forest canopy [22]. Fish experienced three recreated lunar cycles before condition manipulation began (figure 3b).

**Figure 3.**
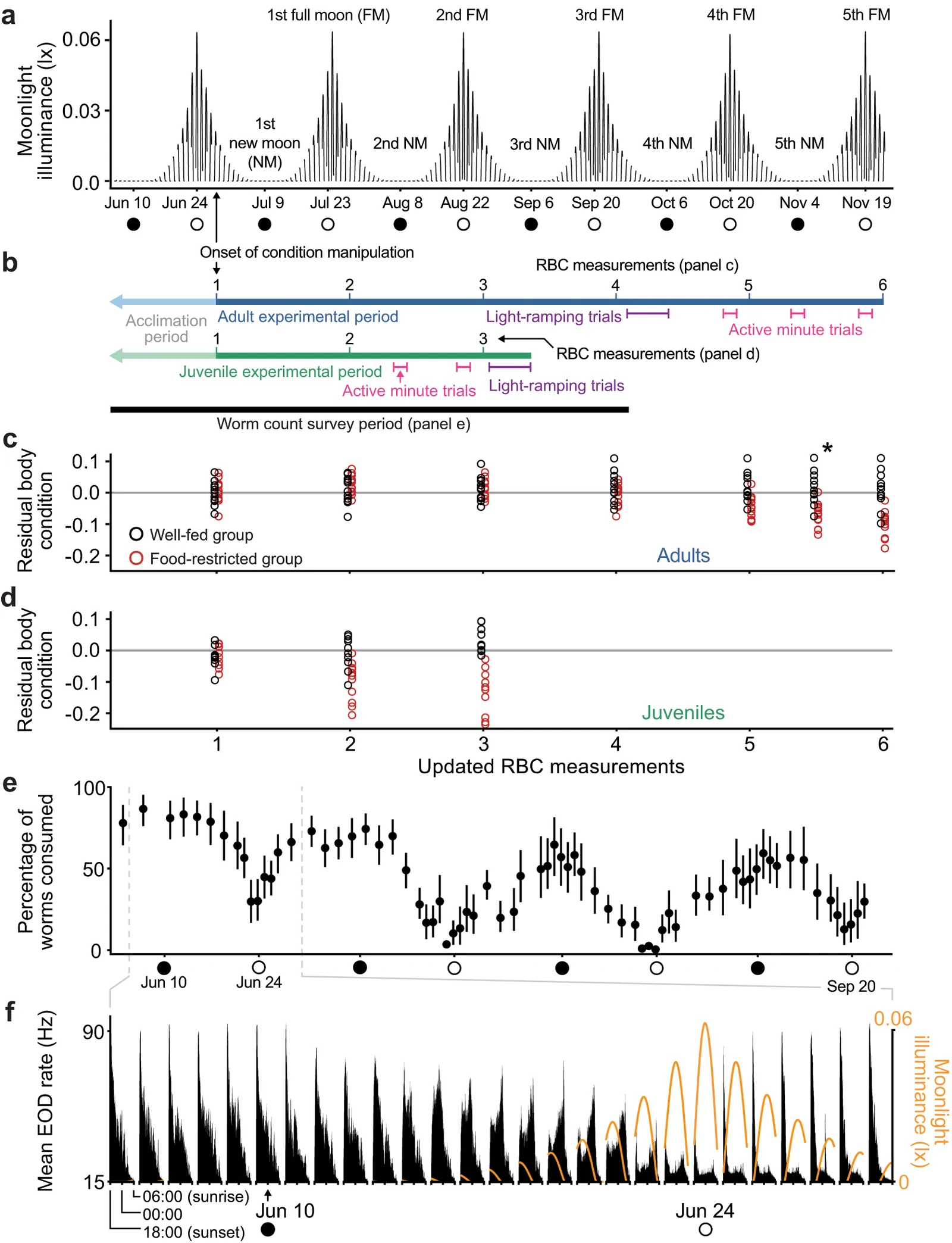
Experimental design for captive sand knifefish, including body-condition manipulation and verification of lunar effects on food consumption and EOD activity. (a) Laboratory-recreated nocturnal moonlight illuminance generated by a *MoonShineP*-controlled LED array, measured at floor level, shown from before the onset of condition manipulation to the end of experiments. (b) Timeline of acclimation, body-condition manipulation in adults (blue bar) and juveniles (green bar), active-minute trials (magenta bars; figure 5), light-ramping trials (purple bars; figure 6) and monitoring of blackworm consumption (black bar). Acclimation began three lunar cycles before the onset of condition manipulation. (c–d) RBC of adults (c) and juveniles (d) throughout the experiment, updated approximately monthly (1 = onset of condition manipulation; 2–6 = subsequent measurements). Well-fed and food-restricted groupings are shown for reference (not used as model predictors). Juveniles reached target RBC range earlier than adults. Asterisk in (c) denotes RBC values linearly interpolated between measurements 5 and 6. (e) Nightly blackworm consumption across 3.5 lunar cycles, showing reduced intake around full moons (means ± 95% CIs; *n* = 23 adults before manipulation; *n* = 11 well-fed adults thereafter). (f) Mean nocturnal EOD rate (18.00–06.00) during a 27-night interval within the worm-consumption survey, beginning five nights before the 10 June new moon. EOD rate is shown in 1-minute bins (each derived from a 15-second recording), averaged across 23 fish; recreated moonlight illuminance (as in panel a) is overlaid.

### 2.8. Captive fish: body-condition manipulation—overview

Newly imported fish initially exhibited high body condition with limited variation. To generate a broader range of body conditions, 12 adults were assigned to a food-restricted group and the remaining 11 to a well-fed group (figure 3c,d). Juveniles were assigned similarly (food-restricted: *n* = 11; well-fed: *n* = 10). Assignments to the feeding groups were random while maintaining similar size ranges across groups and, for adults, similar sex ratios. Feeding groups were used solely to manipulate body condition and were not included as predictors in analyses; instead, RBC was the focal predictor.

### 2.9. Captive fish: body-condition manipulation—quantifying individual maximum food intake

During the approximately three-month acclimation period (figure 3b), fish were fed *ad libitum*, except for 10 moonless nights (centred on the new moon of 11 April) when we quantified maximum food intake for each individual. During this period, fish were offered a pre-counted excess of live oligochaete blackworms (*Lumbriculus variegatus*) in a dish placed in a depression in the sand substrate. Worms were added during the day (10.00–14.00), and uneaten worms were counted the following day. Pre-selected worms of uniform length (2–3 cm) were used throughout, obtained from an in-house laboratory-reared colony. Prior to feeding, worms were rinsed to reduce olfactory cues; because fish did not emerge to forage during daylight hours, they first encountered food after becoming active at night, consistent with their natural foraging pattern.

Maximum nightly food intake (adults: 36–66 worms per fish; juveniles: 13–19 worms per fish) was used to set feeding rations corresponding to 20% (food-restricted) or 100% (well-fed) of each individual's maximum intake. Thus, throughout condition manipulation, rations were individualized for each fish and scaled to its estimated maximum intake. Sand knifefish can forgo feeding for multiple nights during moonlit periods [22], and under the 20% ration, body condition declined gradually. After the 10-night intake-quantification period, all fish were fed at 100% of their individual maximum intake until the start of condition manipulation.

Condition manipulation began on 29 June, when fish began receiving either well-fed (100%) or food-restricted (20%) rations (figure 3b). Food was administered using the same dish-feeding procedure described above.

### 2.10. Captive fish: body-condition manipulation—measurement and monitoring of RBC

RBC was measured at the onset of condition manipulation and monitored thereafter at approximately monthly intervals (figure 3b–d). This interval minimized disturbance associated with handling fish out of water. The manipulation continued until the RBC of the lowest-condition individual in the food-restricted group reached a similar level to the lowest RBC observed in the wild population (figure 2b). Food-restricted adults reached this minimum after approximately five months and juveniles after approximately two months (figure 3b–d). After experiments, fish were euthanized.

### 2.11. Captive fish: verification of lunar effects on food consumption

To test whether moonlight-associated suppression of activity was accompanied by reduced food intake, we conducted a concurrent worm-consumption survey over 3.5 lunar cycles. The survey included all 23 adults before condition manipulation and the 11 well-fed adults thereafter. For each individually housed fish, nightly consumption was calculated from the number of pre-counted worms provided and the number remaining in its feeding dish the following day, while its EOD rate was recorded continuously over the same period to quantify activity (figure 3b,e,f).

### 2.12. Captive fish: behavioural assays of risk-taking—active-minute trials

To quantify the duration of emerged activity, we measured the number of active minutes for each fish on each night. Time spent emerged from the sand, particularly during moonlit periods, is expected to increase exposure to visually oriented predators [22]. Trials were conducted on three bright nights centred on a full moon and three dark nights centred on a new moon (see magenta bars in figure 3b). EOD rate provided a continuously measurable index of activity state: sand knifefish switch from a low resting rate (approx. 10–40 Hz) while buried to a high rate (approx. 70–100 Hz) upon emergence, with little modulation of EOD rate by swimming speed once emerged [24,26]. Because individuals differed slightly in their intrinsic rate ranges, we min–max normalized each fish's EOD rate to 0–1. Minutes with normalized rates ≥0.5 were classified as emerged/active, whereas rates <0.5 were classified as buried/inactive.

We interpreted emerged activity primarily as foraging because sand knifefish continuously scan the substrate after emergence [22–24,26]. This interpretation was further supported by the concurrent worm-consumption survey, in which activity quantified from EOD rate and individually measured food consumption followed the same lunar pattern (figure 3e,f). The principal alternative sources of activity—social and reproductive behaviour—were unlikely to contribute substantially. Fish were housed individually, eliminating direct social interactions; moreover, the species is non-gregarious, non-territorial and non-aggressive [23,31]. Breeding-related activity was also unlikely because none of the subjects had reached full sexual maturity. Under moonlight, active minutes therefore provide an index of time spent foraging under conditions associated with elevated predation risk from visually oriented predators [22].

Before each measurement night, fish in both feeding groups were provided with the food-restricted ration. This standardized food availability during the assay and was intended to minimize short-term hunger differences attributable to the ration provided that day.

### 2.13. Captive fish: behavioural assays of risk-taking—light-ramping trials

To further quantify risk-taking, we used repeated light-ramping trials to measure the illuminance at which active fish burrowed into the sand. The trials were conducted for 10 nights leading up to a new moon (see purple bars in figure 3b), approximately three months after the onset of condition manipulation in adults, and approximately two months in juveniles. We presented a 30-minute linear ramp from 0 to 0.062 lx using *MoonShineP* during the otherwise dark period between 18.30 and 19.00 h. The end-of-ramp illuminance of 0.062 lx corresponds to the maximum reported full-moon illuminance at stream level beneath forest canopy [22]; the ramp therefore remained entirely within the natural lunar range. EOD rate was monitored continuously, and we recorded the illuminance at which min–max normalized rates dropped below 0.5, indicating hiding. Trials in which an individual was inactive at the start of the ramp were excluded, yielding 21 of 23 adults and all 21 juveniles. Fish that did not hide during the ramp were assigned 0.062 lx, the maximum illuminance at the end of the ramp.

### 2.14. Wild and captive fish: statistical analyses

All statistical analyses were performed in R v.4.4.2.

To evaluate size classes in the wild population, we analysed LEA data using a Gaussian finite mixture clustering algorithm (figure 2a) in *mclust*. The fitted first- and second-year length distributions intersected at 108 mm LEA, providing an operational boundary between inferred year classes; fish within the overlapping region could not be classified reliably.

The log_10_(mass) ∼ log_10_(LEA) SMA regression was fitted using *smatr* (model 1). To translate RBC values into percentile ranks within the wild population, we fitted a logistic curve by nonlinear least squares using *minpack.lm* (model 2).

Wild stomach fullness data were analysed using two generalized linear models (GLMs) fitted in *glmmTMB*. The first (model 3) characterized stomach filling over time, with stomach fullness predicted by collection time (log_10_(minutes since the end of nautical twilight)), night category (dark versus bright) and their interaction; this model used a Gaussian distribution with an identity link. The second (model 4) tested, at the population level and irrespective of life stage, whether the relationship between body condition and stomach fullness differed between dark (<0.01 lx) and bright moonlit (>0.03 lx) nights. Stomach fullness was predicted by RBC, night category and their interaction, with collection time included as an additive covariate; this model used a Gaussian distribution with a log link. A constant of 0.0001 was added to stomach fullness before fitting the model to accommodate zero values.

Active minutes on full- and new-moon nights were analysed using generalized linear mixed models (GLMMs) fitted in *glmmTMB*. We used two complementary sets of models to address distinct questions. Our primary analyses used stage-specific models to estimate the relationship between RBC and active minutes within each life stage, moon phase and assay period, using the error distribution supported for each dataset. Our secondary analyses combined adult and juvenile data in separate models for full- and new-moon nights. Each secondary model included an RBC × life stage interaction to compare the adult and juvenile slopes directly under a common model structure.

Each stage-specific model represented a three-night assay period, shown by the magenta bars in figure 3b. Adults were analysed with three such models (models 5A–C): two for full-moon periods (model 5A for the fourth full moon and model 5B for the final (fifth) full moon) and one for the new moon (model 5C; 5th new moon). Juveniles were analysed with two models: one for the full moon (model 6A; final (second) full moon) and one for the new moon (model 6B; 2nd new moon). The distribution family and link function were selected separately for each dataset based on the response distribution and diagnostic checks. Models 5A–C and 6A–B each tested whether active minutes varied with RBC, with individual identity included as a random effect. Because active minutes were totals calculated across entire nights, whereas illuminance varied continuously within moonlit nights, illuminance was not included as a single scalar predictor. Date (night) was included as an additional random effect only in the full-moon models to account for shared variation in moonlight dynamics between successive moonlit nights; new-moon nights were consistently dark. Adult full-moon models used negative binomial type 2 distributions with log links, whereas the juvenile full-moon model used a Poisson distribution with a log link. New-moon models for both life stages used Gaussian distributions with identity links. Consequently, the stage-specific analyses estimated RBC–activity relationships within each moon phase but did not formally compare the RBC slopes between full- and new-moon conditions.

The fourth and fifth adult full-moon assays were analysed separately because condition manipulation continued between them, and the RBC distribution had shifted towards lower values by the fifth full moon. Analysing each assay separately allowed the RBC–activity relationship to be estimated among individuals at the same stage of the manipulation rather than from observations pooled across different stages.

Because sex can influence condition-dependent risk-taking [12], we fitted an exploratory GLMM (model 5D) to the final full-moon adult active-minute data, including an RBC × sex interaction, with individual identity and date treated as random effects (negative binomial type 1 distribution; log link).

For the secondary direct comparisons of RBC–activity slopes between life stages, we fitted separate models combining adult and juvenile data from the final full-moon (model 7A) and final new-moon (model 7B) nights. Each model included an RBC × life stage interaction to compare the adult and juvenile slopes directly. In both models, individual identity was included as a random effect. Date was included as an additional random effect in model 7A. Model 7A used a negative binomial type 1 distribution with a log link, whereas model 7B used a Gaussian distribution with an identity link. Juveniles reached the target RBC range after approximately two months of condition manipulation, whereas adults did so after approximately five months. Accordingly, the models directly compared adult and juvenile slopes after each life stage had reached the target RBC range, although the differing durations of condition manipulation should be considered when interpreting the interaction.

Light-ramping data were analysed using a logistic nonlinear mixed-effects model (NLMM) in *lme4* (model 8). Only adult data were analysed because most juveniles never hid during the light-ramping trials, yielding insufficient variation for modelling. Illuminance at hiding was predicted by RBC, with individual identity included as a random effect on two of the four logistic parameters (upper asymptote and inflexion point). Attempts to include individual identity as a random effect on the remaining parameters failed to converge.

Distribution families for GLMs and GLMMs were selected based on assessment of overdispersion and DHARMa residual diagnostics. For NLMMs, model assumptions were assessed through residual inspection.

## 3. Results

Results are presented first for wild fish and then for captive fish. Full model outputs are provided in tables S1–S13 in the supplementary material, and residual diagnostics for models 1–7B are provided in figures S2–S13 in the supplementary material.

### 3.1. Wild fish: electric organ discharge rate and stomach fullness under moonlight

EOD rate, which distinguishes emerged activity from buried inactivity, declined strongly with increasing moonlight illuminance at the time of observation (figure 4b). On dark nights (<0.01 lx), most recorded fish were active (EOD rate > 70 Hz), whereas on bright nights (>0.03 lx), most were inactive (EOD rate < 40 Hz). Nevertheless, 10 of the 56 fish with usable EOD recordings on bright nights had rates >70 Hz (74.4–91.9 Hz), within the normal range for emerged activity, showing that a minority were active at the time of recording despite bright moonlight. Together, these observations are consistent with strong suppression of nocturnal activity under moonlight, when visual predation risk is expected to be high [22,26].

**Figure 4.**
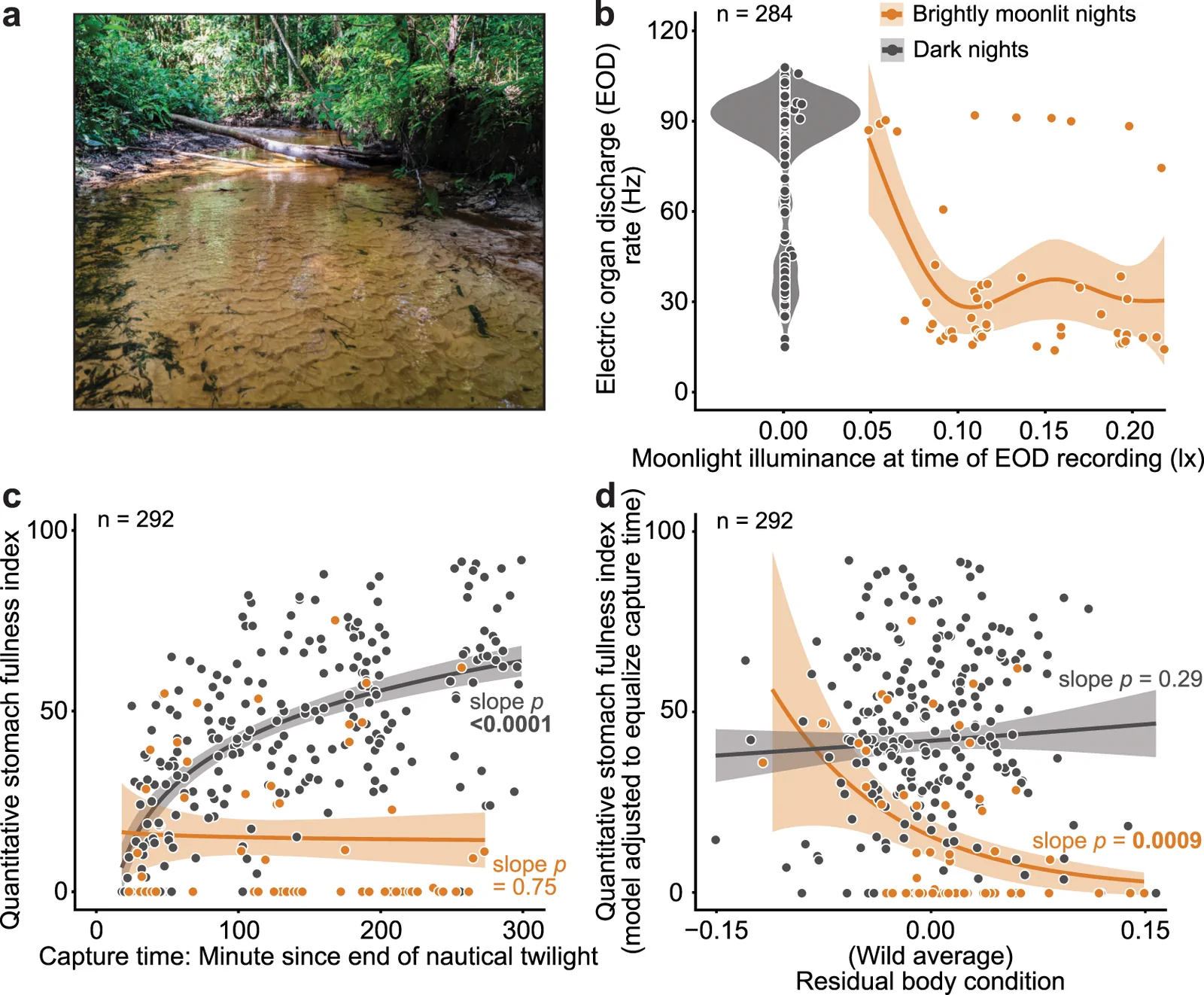
Effects of moonlight on foraging activity and condition dependence in wild sand knifefish. (a) Typical sand-bank habitat of sand knifefish in an Amazon stream. (b) EOD rate recorded immediately before capture versus *MoonShineR*-predicted moonlight illuminance; data modified from fig. 8b of Correa Roldán [42]. Rates below approximately 40 Hz are typically associated with resting while buried in the sand, whereas rates exceeding 70 Hz indicate emerged activity. Dark-night measurements are summarized as a violin plot. For bright-night measurements, the orange line and shaded band show a descriptive smoother fitted using a generalized additive model (GAM), with its 95% CI. The GAM was used for visualization only and not for statistical inference. (c) Stomach fullness versus capture time (minutes since the end of nautical twilight) on dark nights (illuminance < 0.01 lx) and bright nights (full moon and preceding three nights; illuminance 0.03–0.22 lx). (d) Stomach fullness versus RBC on dark and bright moonlit nights. In this panel, points and regressions are plotted with stomach fullness adjusted to a standardized capture time (112 minutes after nautical twilight), but the underlying model was based on the unadjusted stomach-fullness measurements, with capture time included as a covariate. In (c,d), lines show GLM predictions. Shaded areas show 95% CIs, and points are individuals; bold *p*-values indicate significant slopes (*p* < 0.05).

Stomach fullness (QSF; see §2.2) increased through the night on dark nights, rising logarithmically with time since the end of nautical twilight (hereafter ‘nightfall’) (model 3, GLM, *p* < 0.0001, *z* = 11.40; figure 4c). Because QSF is calculated as stomach-content mass divided by the combined mass of the stomach and its contents, this bounded ratio inherently produces diminishing increases as stomach contents accumulate. By contrast, QSF showed no detectable relationship with time since nightfall on bright nights (*p* = 0.75, *z* = −0.32), and most individuals had zero fullness regardless of sampling time. Direct comparisons showed that the time-fullness slope was significantly weaker on bright than on dark nights (time × night-category interaction: *z* = −5.48, *p* < 0.0001). Together, these results are consistent with strong suppression of foraging under moonlight.

### 3.2. Wild fish: condition dependence of stomach fullness under moonlight

Although 31 of 59 individuals had empty stomachs on bright nights, 28 (47.5%) showed non-zero stomach fullness, including some with high fullness (figure 4d), raising the question of whether foraging under moonlight was associated with body condition. After accounting for collection time, the RBC–fullness slope was significantly more negative on bright than on dark nights (model 4, GLM, RBC × night-category interaction: *p* = 0.0005; *z* = −3.46). Stomach fullness on bright nights was negatively related to RBC, with lower-RBC individuals showing higher stomach fullness (*p* = 0.0009, *z* = −3.33; figure 4d). By contrast, RBC did not predict stomach fullness on dark nights (*p* = 0.29, *z* = 1.06). We did not test whether the relationship between RBC and stomach fullness differed between life stages in the wild dataset because 23 of the 59 bright-night individuals had lengths within the overlap between the first- and second-year length distributions and therefore could not be assigned reliably to a life stage (figure 2a). Model 4 therefore estimated the population-level relationship between RBC and stomach fullness irrespective of life stage.

The negative effect of RBC on stomach fullness during bright nights was driven largely by individuals at the extremes of body condition: most low-condition fish had non-zero stomach fullness, whereas most high-condition fish had zero fullness (figure 4d). Because individuals in extreme body condition are uncommon in the population (figure 2c), they contribute substantial leverage to the regression. Nonetheless, their fullness values reflect biologically meaningful behaviour.

### 3.3. Captive fish: lunar modulation of electric organ discharge rate and food consumption

Captive sand knifefish exhibited a pronounced lunar rhythm in activity under the recreated moonlight regime, with EOD rate progressively suppressed towards full moon and elevated towards new moon (figure 3f). During the overlapping 27-night interval, worm consumption followed the same lunar trajectory, declining towards full moon and increasing again towards new moon (figure 3e,f). Because food consumption was measured separately and concurrently with EOD rate, this concordance shows that moonlight-associated suppression of activity was accompanied by reduced feeding.

### 3.4. Captive fish: behavioural assays of risk-taking—active-minute trials

In the primary, stage-specific models, adult activity on full-moon nights varied with body condition. Adults with lower RBC remained active for longer than those with higher RBC during both the 4th full moon (model 5A, *p* = 0.011, *z* = −2.55; figure 5a) and the final, 5th full moon (model 5B, *p* = 0.0003, *z* = −3.59; figure 5c). As body condition declined between these assay periods—the lowest RBC decreased from −0.092 (percentile rank of 3.2 in the wild distribution; figure 5a) to −0.177 (percentile rank of 0.1; figure 5c)—the relationship became more apparent. An RBC of −0.177 corresponds to a 33.5% reduction in mass relative to the wild average (RBC = 0). At the final full moon, predicted active time increased exponentially with declining RBC, from little or no activity at RBC > 0 to approximately 26 minutes at RBC = −0.092 and 57 minutes at RBC = −0.177 (figure 5c).

**Figure 5.**
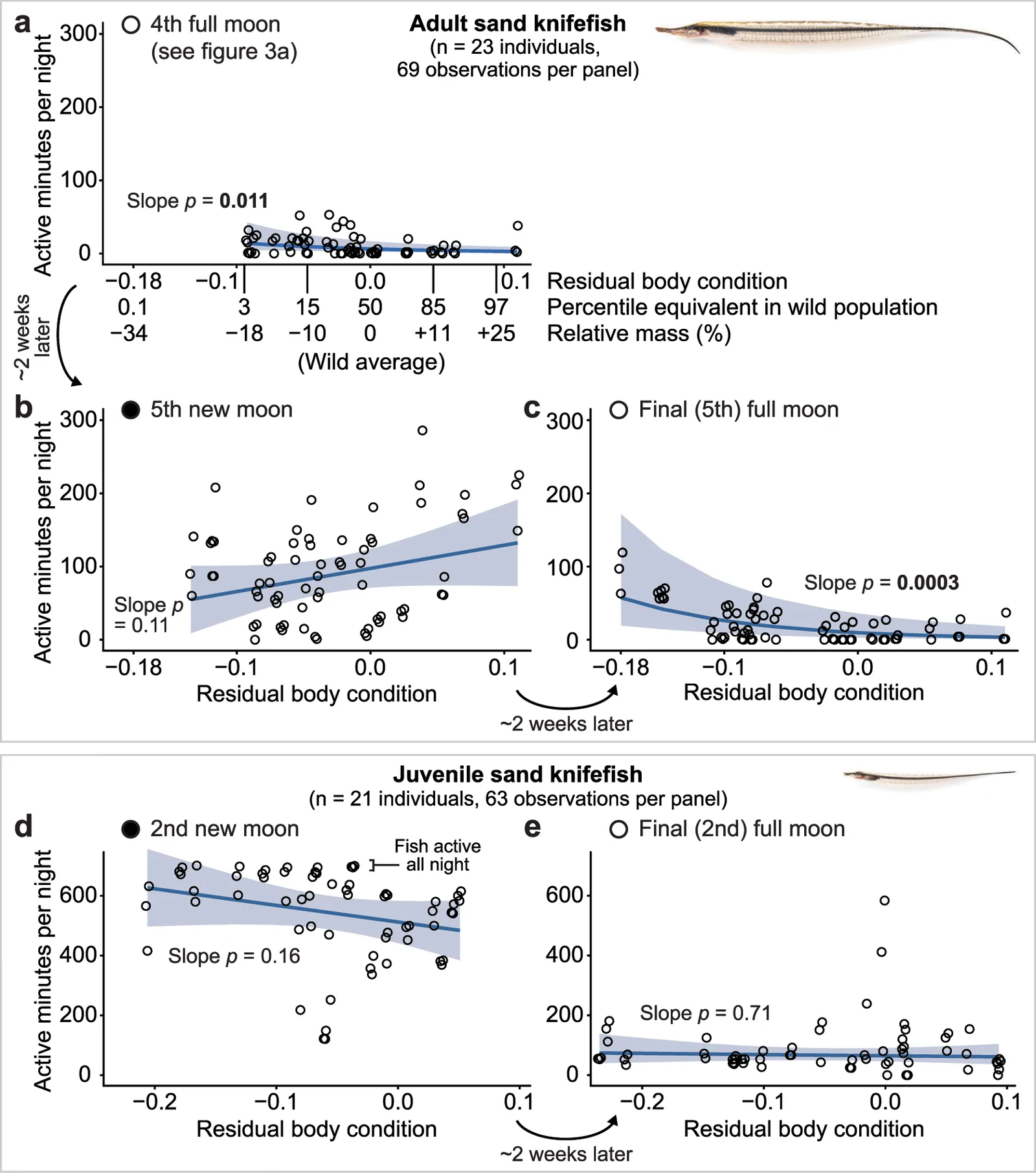
Effects of RBC on active minutes under laboratory-recreated new- and full-moon conditions. (a–c) Adults: 4th full moon (a), subsequent (5th) new moon (b), and final (5th) full moon (c) (figure 3a–c). (d–e) Juveniles: 2nd new moon (d) and final (2nd) full moon (e) (figure 3a,b,d). Each point represents one individual on one night; each panel pools three nights centred on the focal new-moon (NM) or full-moon (FM) night. RBC values use the nearest measurement shown in figure 3c–d; for the 5th new moon (b), RBC was linearly interpolated between measurements 5 and 6. Points are slightly jittered along the x-axis. Lines show predictions and shaded areas show 95% CIs from stage-specific GLMMs fitted separately to each panel (fixed effects only). Comparisons of predicted activity levels across life stage panels are descriptive. A secondary interaction model, not depicted here, directly compared the adult and juvenile RBC slopes in the final full-moon assays (c and e) and detected a significant RBC × life stage interaction (model 7A). The corresponding new-moon interaction model (model 7B) did not satisfy residual assumptions and was not used for between-stage inference. Bold *p*-values indicate significant RBC slopes (*p* < 0.05). In (a), the secondary x-axes show wild-population percentile equivalents (figure 2c) and relative mass (% deviation from expected).

During new-moon nights, when moonlight was absent and light-associated predation risk was expected to be low, adult active minutes were not detectably related to RBC (model 5C, *p* = 0.11, *z* = 1.62; figure 5b), and overall activity was substantially higher than during full moons.

The exploratory RBC × sex model provided no evidence that activity differed between sexes at RBC = 0 (model 5D; *p* = 0.089, *z* = −1.70) or that the RBC–activity slope differed between sexes (*p* = 0.145, *z* = 1.46).

Juvenile active minutes were not detectably related to RBC on either full-moon (model 6A; *p* = 0.71, *z* = −0.38; figure 5e) or new-moon nights (model 6B; *p* = 0.16, z = −1.40; figure 5d).

The secondary full-moon interaction model directly compared the adult and juvenile RBC–activity slopes. On the log-link scale, the estimated slope was −7.45 in adults (*p* = 0.002, *z* = −3.17) and −1.25 in juveniles (*p* = 0.387, *z* = −0.87). The adult slope was significantly more negative than the juvenile slope (model 7A, RBC × life stage interaction: estimated adult–juvenile slope difference = −6.20, s.e. = 2.76, *p* = 0.025, *z* = −2.25). Thus, the increase in activity associated with lower RBC was significantly stronger in adults than in juveniles during the final full-moon nights. Each life stage was assayed after reaching the target RBC range, although the duration of condition manipulation differed between stages; this design difference should be considered when interpreting the interaction.

The RBC × life stage interaction in the corresponding new-moon model did not reach statistical significance at *α* = 0.05 (model 7B; estimated adult–juvenile slope difference = 871.89, *z* = 1.93, nominal *p* = 0.054). However, diagnostics indicated non-random patterns in the residuals, unequal variance between life stages, and a significant outlier test (supplementary material, figure S13). We therefore draw no firm conclusion about whether the new-moon slopes differed between life stages.

Juveniles were also generally more active than adults on both full- and new-moon nights. At RBC = 0, the stage-specific models estimated 512 minutes (8.5 hours) of activity for juveniles and 103 minutes (1.7 hours) for adults on new-moon nights (figure 5b,d); the corresponding estimates on full-moon nights were approximately 64 and 9 minutes, respectively (figure 5c,e). Because these estimates were generated by separate stage-specific models, they are presented descriptively rather than as a formal test of baseline life stage differences.

### 3.5. Captive fish: behavioural assays of risk-taking—light-ramping trials

In the light-ramping trials, the illuminance at which adult sand knifefish hid by burrowing into the sand varied with body condition. In a logistic NLMM (model 8), low-condition adults continued to emit the high EOD rates diagnostic of the emerged, active state until illuminance reached approximately 0.04 lx, whereas high-condition adults hid at much lower illuminance (approx. 0.003 lx; figure 6a). The 95% confidence intervals for the upper and lower asymptotes did not overlap (upper: 0.0346–0.0477 lx; lower: 0.0006–0.0051 lx), supporting separation between the estimated asymptotes. All four fixed-effect parameter estimates had | *t* | > 2. Illuminance at hiding followed a threshold-like pattern, beginning to increase near RBC = 0, with the steepest change occurring at RBC ≈ −0.017 (figure 6a).

**Figure 6.**
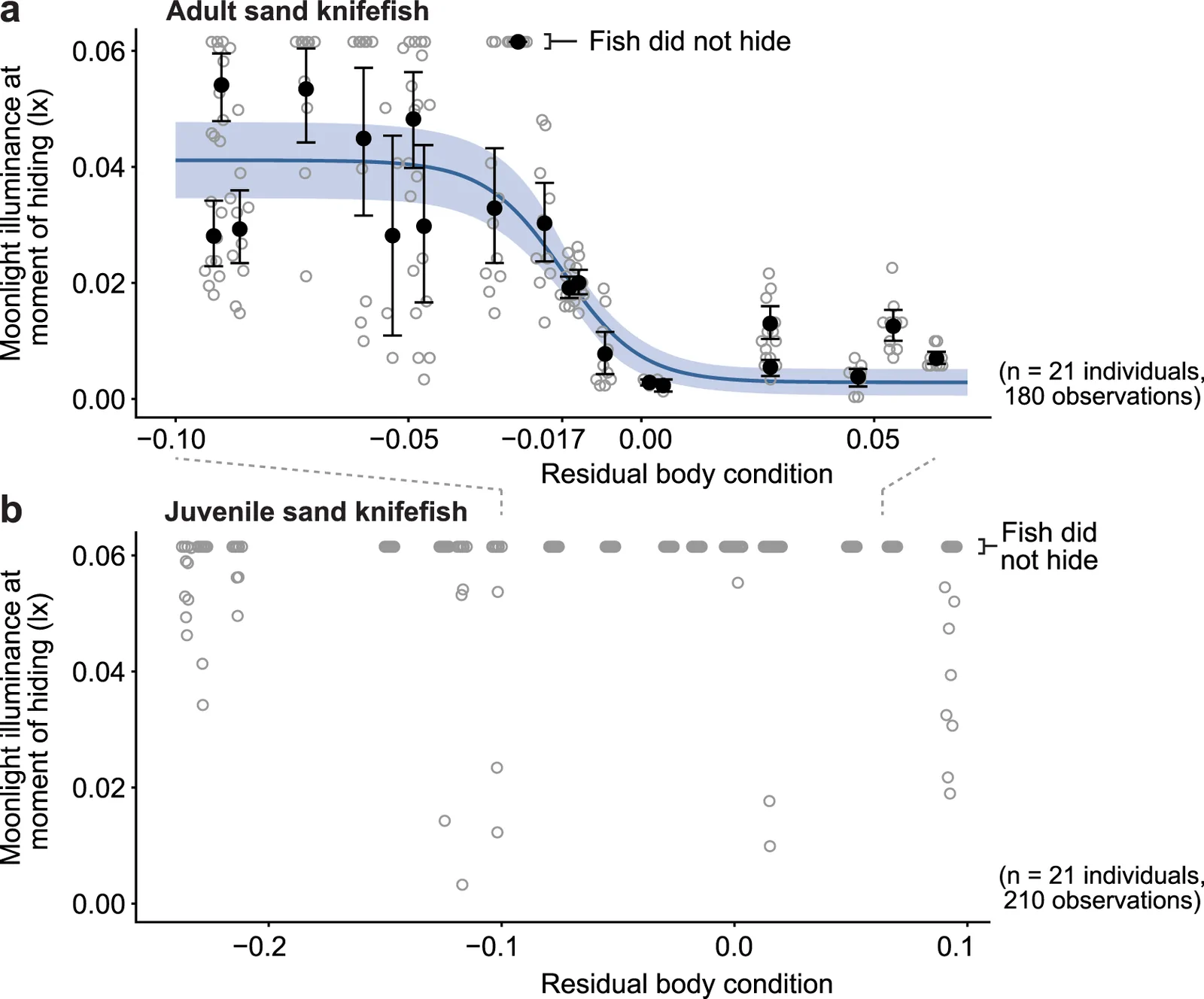
Relationship between RBC and illuminance at hiding during laboratory light-ramping trials. (a) Adults: illuminance at the moment of hiding (sand-burrowing) during 30-minute light ramps. Each open grey point represents one trial; black points show individual means ± s.e. The solid line and shaded area show the fitted relationship and 95% CIs, respectively, from a logistic nonlinear mixed-effects model (NLMM; fixed effects only). (b) Juveniles: each open grey point represents one trial. No model was fitted because juveniles did not hide in most trials, leaving insufficient variation; the data are shown descriptively. In both panels, trials in which fish did not hide were assigned the maximum illuminance reached at the end of the ramp (0.062 lx). Points are slightly jittered along the x-axis.

By contrast, juveniles rarely hid during the light-ramping trials, remaining active as light-associated predation risk increased. Most were still active even at the maximum illuminance (0.062 lx; figure 6b). The rarity of hiding provided insufficient variation to model the relationship between RBC and illuminance at hiding; among the few juvenile trials in which hiding occurred, illuminance at hiding showed no obvious relationship with RBC.

## 4. Discussion

We tested whether body condition predicted foraging under predation risk in wild sand knifefish and in separate captive juvenile and adult groups under controlled conditions. In the wild, lower-RBC individuals had higher stomach fullness on bright moonlit nights (higher risk), whereas RBC did not predict stomach fullness on dark nights (lower risk). In captive adults, lower RBC predicted longer activity on full-moon nights and hiding at higher illuminance, but no association with activity was detected on new-moon nights. In captive juveniles, RBC did not detectably predict activity on either full- or new-moon nights, and most juveniles remained active throughout the light-ramping trials, leaving insufficient variation for modelling. A secondary interaction analysis showed that on full-moon nights, activity increased more strongly with declining RBC in adults than in juveniles. Overall, our findings support condition-dependent risk-taking in adults, whereas no corresponding relationship was detected in juveniles.

### 4.1. Asset protection predictions across life stages

The secondary interaction analysis of the final full-moon nights (model 7A) showed a significant adult–juvenile slope contrast, consistent with our prediction of stronger condition dependence in adults, although we cannot exclude some contribution from the differing durations of condition manipulation.

The adult pattern is consistent with the asset protection principle and life-history theory. Under asset protection, individuals with greater residual reproductive value—often those in high body condition—should be relatively risk-averse, whereas individuals in low condition should accept greater risk to recover energetic reserves. Adult sand knifefish followed this expectation: lower condition predicted greater risk-taking under moonlight. This is consistent with the strong time constraints to improve energetic state imposed by the species' short-lived life history, in which reproduction is largely confined to a single terminal breeding season, placing the species towards the semelparous end of the iteroparity–semelparity continuum. Such constraints should be less pronounced in longer-lived species with repeated breeding opportunities, in which individuals in low condition can defer reproduction and recover reserves before breeding [4,18].

The juvenile active-minute results were also consistent with our *a priori* prediction of weak or undetectable condition-dependent modulation of risk-taking: RBC did not detectably predict active minutes on either full- or new-moon nights. In many species—particularly in high-predation or unstable environments—low-condition juveniles may accept elevated predation risk to accelerate growth because survival increases sharply after individuals exceed a critical size [4,41]. In sand knifefish, however, high juvenile recapture rates suggest low baseline mortality [31], which should reduce the fitness advantage of accepting additional risk to accelerate growth [36]. The absence of detectable condition dependence in juvenile active minutes is consistent with this interpretation, although it does not identify the underlying mechanism.

Comparisons with other taxa illustrate how different mechanisms can alter the direction of life stage patterns in condition-dependent foraging. In porcupines, young individuals increased their use of risky but rewarding open areas during winter nutritional stress, whereas adults used these areas more consistently throughout winter [21]. Sweitzer & Berger attributed this contrast to size-related vulnerability: smaller young were more vulnerable to predators, so the same habitat did not impose equivalent predation risk across life stages. In ground squirrels, juveniles receiving low-energy food increased foraging and remained less vigilant, whereas juveniles receiving high-energy food increased vigilance and reduced foraging, making their behaviour more similar to that of adults [20]. Arenz and Leger attributed this response to the energetic demands of juvenile growth.

Our finding that condition-dependent risk-taking was significantly stronger in adults than in juveniles under elevated predation risk contrasts with the overall pattern reported in a meta-analysis spanning fishes, birds, mammals and insects, but is consistent with the reproductive time constraints faced by adults approaching a terminal breeding season [4,12,16]. Together, these findings show that life stage patterns in condition-dependent foraging need not follow a consistent direction. Because the juvenile-to-adult transition entails multiple correlated physiological and ecological changes, such patterns cannot readily be attributed to a single mechanism. Their expression instead depends on how reproductive time constraints, growth-related energetic demand and size-related vulnerability alter the energetic gain–safety trade-off within particular ecological and life-history contexts.

### 4.2. Adult risk-taking responses to variation in RBC

The active-minute and light-ramping trials captured complementary aspects of condition-dependent risk-taking: the former measured the total nightly duration of activity under a naturalistic moonlight regime, whereas the latter measured the illuminance at which fish hid during a 30-minute increase in light—a behavioural response that previous field and laboratory studies have associated with moonlight-mediated predation risk [22,26].

In captive adults, declining RBC was associated with an exponential increase in foraging activity on full-moon nights (figure 5c). Individuals in low condition—particularly those in the lowest percentiles of the wild RBC distribution (figure 2c)—remained active far longer than those in high condition, indicating a sharp increase in risk-taking as energetic state declined. Assuming that time spent foraging under moonlight scales with both predation exposure and energetic gain, this exponential pattern suggests a disproportionate decline in the valuation of safety as body condition worsens. Individuals in the lower tail of the wild RBC distribution had substantial energetic deficits relative to the wild average, and their risk-prone foraging behaviour is therefore consistent with an effort to recover condition and thereby improve future reproductive potential.

In the light-ramping trials, the modelled relationship between RBC and illuminance at hiding was logistic (figure 6a). As RBC fell below the wild average, adults remained active until markedly higher illuminance levels were reached, with the steepest increase occurring at RBC ≈ −0.017—a pattern indicating a threshold-like shift in risk-taking. Unlike active minutes, which continued to rise towards the lowest RBC values, the illuminance required to elicit hiding rose rapidly over a narrow RBC range and then approached an asymptote. This suggests that relatively modest declines in condition are sufficient to promote risk-taking, but that even low-condition adults showed an apparent upper illuminance threshold beyond which they hid. In our light-ramping trials, this estimated upper bound was approx. 0.04 lx, close to the maximum full-moon illuminance beneath the forest canopy [22]. This logistic relationship is consistent with state-dependent models in which behavioural thresholds shift nonlinearly with internal state, often following sigmoidal fitness functions [48,49].

### 4.3. Juvenile risk-taking responses to variation in residual body condition

Although juvenile activity did not show detectable RBC dependence on either full- or new-moon nights, juveniles were generally highly active. In the light-ramping trials, most juveniles remained active even at the maximum illuminance, whereas adults usually hid before the end of the ramp. This apparent between-stage difference in light-ramping response could not be tested formally because hiding was rare among juveniles.

The generally high activity of juveniles may reflect greater baseline energetic demand: mass-specific metabolic rate is greater early in life, and growth requires sustained energy intake [50]. A second, more speculative possibility is that juveniles have lower foraging efficiency—perhaps owing to weaker swimming performance, less effective excavation of buried prey or a shorter electrosensory detection range—requiring longer foraging to meet energetic demands.

The absence of a detectable relationship between RBC and active minutes in juveniles raises the question of whether low-condition juveniles in the wild can recover energetic deficits later in life, and under what circumstances—for example, during the rainy season, when food availability increases [32] and predation risk from visually oriented predators may decline with increased stream volume and turbidity [51]. Compensatory growth after a period of growth arrest can allow individuals to catch up to growth targets [40]. However, energetic deficits early in life can also impose long-term, sometimes irreversible, physiological damage [52]. Whether sand knifefish juveniles exhibit compensatory growth, and whether early deficits carry persistent costs, remains unknown.

### 4.4. Limitations and complementary evidence from wild and captive data

The field stomach-fullness analysis was conducted at the population level and did not test whether the relationship between RBC and stomach fullness differed between juveniles and adults. Two features of the field dataset made a reliable stage-specific analysis difficult. First, only 59 fish were sampled on bright nights, and individuals in the lower tail of the RBC distribution—those most likely to exhibit condition-dependent risk-taking—were uncommon. Second, life stages were less clearly separable in the wild than in captivity: 23 of the 59 bright-night individuals fell within the overlap between the first- and second-year length distributions (figure 2a), making life stage assignment uncertain for a substantial portion of the sample. Logistical constraints prevented us from extending sampling across additional lunar cycles to increase the bright-night sample available for stage-specific analysis. Accordingly, the field analysis does not provide evidence either for or against a life stage difference in condition dependence.

More generally, stomach fullness provides a temporally limited snapshot of recent feeding rather than a direct measure of foraging duration. Feeding success can vary stochastically among individuals in the wild, and fullness at capture depends on collection time: fish captured later in the night had more time to accumulate stomach contents, and we accounted for this variation by including collection time as a covariate. In addition, stomach-content mass is difficult to measure precisely when food amounts are small.

The controlled experiments addressed several of these limitations. We used clearly separated size classes—relatively young juveniles and large late second-year adults—and excluded individuals in the transitional size range between them; behaviour in this intermediate size range, therefore, remains unknown. Body condition was manipulated across the range observed in the wild, and individual activity was monitored continuously under quantitatively recreated lunar illumination.

Interpretation of our results necessarily relies on indirect measures because predation and foraging effort are difficult to quantify directly. Moonlight provides an index of visual predation risk, EOD rate identifies emerged activity and stomach fullness indexes recent feeding; however, we did not directly observe predation events or measure foraging duration. Moonlight also does not encompass risk from predators using other sensory modalities. Electric eels (*Electrophorus*), for example, are likely predators of *G. rondoni*, with signalling interactions between them consistent with mutual detection of their electric signals by passive electroreception [53]. Our lunar manipulation therefore addresses only the visually mediated component of predation risk. Although we recreated lunar illumination within the natural range observed in rainforest streams, the experimental design cannot exclude every physiological effect of light. Nevertheless, the rapid reversibility of moonlight-induced hiding [22,26], the association between moonlight responses and eye size among co-occurring species [22] and the concordant lunar trajectories of emerged activity and food consumption (figure 3e,f) support the antipredator-foraging interpretation over a generalized suppression of activity unrelated to predation and feeding.

The feeding manipulation likewise cannot separate body condition from all motivational consequences of sustained energetic deficit. Providing both feeding groups with the food-restricted ration before each measurement night standardized food availability during the assay and likely minimized short-term differences in hunger attributable to that day's ration, but could not eliminate differences arising from their longer-term feeding histories; behavioural associations with RBC should therefore be interpreted in that context.

Despite these limitations, the field and laboratory approaches combined population-level evidence of condition-dependent foraging in the wild with controlled tests of RBC–activity relationships within each captive life stage. The secondary full-moon interaction analysis additionally provided the formal adult–juvenile comparison that the field data could not support.

## 5. Conclusion

Our study found a clear life stage difference in condition-dependent risk-taking in the sand knifefish, with foraging on full-moon nights increasing more strongly in adults than in juveniles as body condition declined. Adults exhibited strong condition-dependent risk-taking, with low-condition individuals accepting greater moonlight-associated predation risk than high-condition individuals. By contrast, although juveniles were generally more active than adults, their nightly foraging activity did not show detectable RBC dependence on either full- or new-moon nights. The adult pattern is consistent with increased foraging to mitigate energetic deficits under the strong time constraints preceding a single terminal late-life breeding season. The juvenile pattern is consistent with high juvenile recapture rates suggesting low baseline mortality and, consequently, a smaller fitness advantage of accepting additional predation risk. These findings were obtained using clear classification of captive juveniles and adults based on size and maturation status, ecologically realistic recreation of natural moonlight dynamics and a body-condition metric quantified relative to the wild population. The stronger condition dependence in adults contrasts with the more common pattern reported in a recent meta-analysis [12], illustrating how species-specific life histories can shape the expression of state-dependent behavioural trade-offs across life stages.

## Supplementary Material



## Data Availability

All data presented in this manuscript are available via the Dryad Digital Repository [54]. Supplementary material is available online [55].
