# Supplementary material for: Life stage shapes condition-dependent foraging under predation risk in an electric fish
Source: R Soc Open Sci. 2026 Oct 21;13(10):260914. doi: 10.1098/rsos.260914 (PMC13682893; doi:10.1098/rsos.260914)
Supplement: Supplementary file 1 [file rsos.260914.zip › rsos.260914/rsos260914_si_001.docx]

**Supplementary Material for “Life stage shapes condition-dependent foraging under predation risk in an electric fish”**

Lok Poon^1^ & William G.R. Crampton^1*^

^1^ Department of Biology, University of Central Florida, 4100 Libra Dr., Orlando, Florida, 32816, USA

* Correspondence: crampton@ucf.edu

**Figure S1. Electric organ discharge (EOD) recording tanks and electrode setup. (a)** Experimental chamber under a 12:12 h light–dark cycle with a simulated moonlight rhythm. The room was centrally heated by an overhead electric heater, and air was circulated by a fan to prevent thermal stratification. Light levels were regulated using a Lutron Grafik Eye control system. Moonlight was simulated using the MoonShineP system. The red circle indicates the sensor of the ILT5000 low-light meter. Each 20-gallon tank housed one sand knifefish. The upper rack was lined with light-blocking paper so that the top and bottom shelves received similar light levels. (b) Each tank contained an array of four orthogonally arranged recording electrode pairs (e.g., 1 and 1’). Signals from the electrode pairs were rectified, summed, and amplified by custom electronics in an adjacent control room to produce a single output signal, which was digitised in real time using a LabVIEW program. This configuration allowed EOD detection regardless of fish orientation. This recording setup follows Poon & Crampton [1]. (c) Electrode array in an operating tank with a sand bottom and an air-driven sponge filter (back left). The nickel–chromium electrodes are covered with acrylic sponge to prevent the fish from touching them.

**Model 1:** Wild sand knifefish SMA regression between body mass and length to end of anal-fin terminus (LEA)

Standardised major axis (SMA) regression fitted using package *smatr* (R syntax):

*log_10__transformed_mass ~ log_10__transformed_LEA*

Variables:

- log_10__transformed_mass (response) = log_10_-transformed mass (g) (continuous)
- log_10__transformed_ LEA (predictor) = log_10_-transformed length to end of anal-fin terminus (LEA) (mm) (continuous)

**Table S1. Model 1 output.** Significant *P*-values in bold.

|  | ***Estimate*** | ***Upper 95% CI*** | ***Lower 95% CI*** | ***P-value*** |
| --- | --- | --- | --- | --- |
| Intercept | -5.817 | -5.714 | -5.920 | — |
| Slope | 2.973 | 3.025 | 2.922 | **< 2e-16** |

N = 292

R^2^ = 0.978

**Figure S2. Diagnostic plots of residuals versus fitted values for Model 1.** Visual inspection indicates no evident heteroscedasticity.
**Model 2:** Wild sand knifefish percentile predicted by residual body condition

Logistic curve fitted by nonlinear least squares using package *minpack.lm* (R syntax):

*percentile ~ 1 / (1 + exp(-k * (residual_condition - i)))*

Variables:

- percentile (response) = the individual’s percentile rank within the wild population (continuous)
- residual_condition (predictor) = residual body condition (continuous)

Parameters:

- k = slope
- i = inflection point
- The upper asymptote is fixed to 1 (the numerator)

Note:

This exploratory model was used only to convert RBC to an approximate percentile rank within the wild population.

**Table S2. Model 2 output.** Significant *P*-values in bold.

|  | **Estimate** | **SE** | ***t*-value** | ***P-*value** |
| --- | --- | --- | --- | --- |
| k (slope) | 37.529 | 0.181 | 207.12 | — |
| I (inflection point) | -0.0015 | 0.00012 | -12.46 | **< 2e-16** |

N = 292

**Figure S3. Diagnostic plots for Model 2 residuals.** The residual-versus-fitted plot shows systematic structure, and the Q–Q plot departs from normality in the lower tail. Model 2 is therefore interpreted only as an approximate, exploratory conversion from RBC to percentile rank.

**Model 3:** Effect of time since nightfall on stomach fullness in wild sand knifefish

Generalised linear model (GLM) fitted using package *glmmTMB* (R syntax):

Distribution family: Gaussian, identity link

*stomach_fullness* ~ *log10(time_since_nightfall) * dark_bright_night*

Variables:

- stomach fullness (response) = quantitative stomach fullness index (QSF): mass of stomach contents divided by the combined mass of the stomach and its contents (continuous).
- log_10_(time_since_nightfall) (predictor) = the collection time of the individual expressed as the number of minutes since the end of nautical twilight (continuous). log_10_-transformed because increase in stomach fullness slows down over time given how QSF was calculated.
- dark_bright_night (predictor) = whether the night of collection was “dark” or “bright” (categorical), see definitions in main text, Methods.

**Table S3. Model 3 output.** Significant *P*-values in bold.

| **Effect type** | **Term** | **Estimate** | **SE** | ***z*-value** | ***P*-value** |
| --- | --- | --- | --- | --- | --- |
| Fixed | *(Intercept) (reference = dark)* | -52.696 | 8.508 | -6.194 | **5.86e-10** |
| Fixed | *log10(time_since_nightfall)* | 46.949 | 4.117 | 11.403 | **< 2e-16** |
| Fixed | *dark_bright_night (bright)* | 72.900 | 18.822 | 3.873 | **0.0001** |
| Fixed (interaction term) | *dark_bright_night (bright):*  *log10(time_since_nightfall)* | -49.502 | 9.031 | -5.481 | **4.23e-08** |

N = 292

Adjusted R^2^ = 0.433

Note: With bright nights as the reference level, the log_10_(time_since_nightfall) slope was −2.553 ($z=-0.32$, $P=0.75$).

**Figure S4. DHARMa diagnostic plots for Model 3.** The plots assess residual uniformity (Q-Q), residuals versus predicted values, within-group deviations from uniformity (dark versus bright nights), and dispersion. Overall, diagnostics indicated no major violations. A deviation from within-group uniformity was detected for the bright-night group, consistent with a higher frequency of zero stomach fullness values (empty stomachs) on bright nights.

**Model 4:** Effect of residual body condition on stomach fullness in wild sand knifefish

Generalised linear model (GLM) fitted using package *glmmTMB* (R syntax):

Distribution family: Gaussian, log link

*stomach_fullness +* 0.0001 ~ *residual_condition * dark_bright_night + log10(time_since_nightfall)*

Variables:

- stomach fullness (response) = quantitative stomach fullness index (QSF): mass of stomach contents divided by the combined mass of the stomach and its contents (continuous). A small constant of 0.0001 was added to allow the log link.
- log_10_(time_since_nightfall) (predictor) = the collection time of the individual expressed as the number of minutes since the end of nautical twilight (continuous). Log_10_-transformed because the increase in stomach fullness slows down over time given how QSF was calculated.
- dark_bright_night (predictor) = whether the night of collection was “dark” or “bright” (categorical), see definitions in main text, Methods.
- residual_condition (covariate) = the residual body condition of each individual (continuous).

**Table S4. Model 4 output.** Significant *P*-values in bold.

| **Effect type** | **Term** | **Estimate** | **SE** | ***z*-value** | | ***P*-value** |
| --- | --- | --- | --- | --- | --- | --- |
| Fixed | *(Intercept) (reference = dark)* | 1.550 | 0.246 | 6.290 | **3.18e-10** | |
| Fixed | *residual_condition* | 0.660 | 0.621 | 1.063 | 0.288 | |
| Fixed | *dark_bright_night (bright)* | -1.153 | 0.194 | -5.941 | **2.83e-09** | |
| Fixed (interaction term) | *dark_bright_night (bright):*  *residual_condition* | -12.453 | 3.595 | -3.464 | **0.000533** | |
| Fixed (covariate) | *log10(time_since_nightfall)* | 1.065 | 0.111 | 9.583 | **< 2e-16** | |

N = 292

Adjusted R^2^ = 0.427

**Figure S5. DHARMa diagnostic plots for Model 4.** The plots assess residual uniformity (Q–Q), residuals versus predicted values, within-group deviations from uniformity (dark versus bright nights), and dispersion. Overall, diagnostics indicated no major violations; within-group deviation from uniformity and the Levene test for homogeneity of variance were non-significant.

**Model 5A:** Active-minute experiment: Captive adult sand knifefish active minutes per night predicted by residual body condition on the 4^th^ (second-last) full moon

Generalised linear mixed-effects model (GLMM) fitted using package *glmmTMB* (R syntax):

Distribution family: negative binomial (nbinom2; *glmmTMB* terminology), log link

*active_minute ~ residual_condition + (1|fish_identity) + (1|date)*

Variables:

- active_minute (response) = number of active minutes within the observation night (count)
- residual_condition (predictor) = the residual body condition measurement most recent to the test date (continuous)
- fish_identity (random effect) = individual identification number (categorical)
- date (random effect) = date of the measurement (categorical)

Note:

Date was included as a random effect in all full-moon models (Models 5A, 5B, 5D, 6A and 7A). Active minutes were quantified for three nights centred on the full moon (FM−1, FM, FM+1). Moonlight conditions shift across these nights, producing a systematic date effect. For example, on the night of the full moon, the entire night was moonlit, whereas on the nights before and after there are dark periods before moonrise and after moonset, respectively [1]. In contrast, models using data collected around the new moon do not exhibit this date structure and therefore did not require date as a random effect.

**Table S5. Model 5A output.** Significant *P*-values in bold.

| **Effect type** | **Term** | **Estimate** | **SE** | ***z*-value** | **SD** | ***P*-value** |
| --- | --- | --- | --- | --- | --- | --- |
| Fixed | *(Intercept)* | 1.762 | 0.523 | 3.367 | NA | **0.00076** |
| Fixed | *residual_condition* | -9.089 | 3.568 | -2.547 | NA | **0.0109** |
| Random | *fish_identity* | — | — | — | 0.561 | — |
| Random | *date* | — | — | — | 0.822 | — |

N (fish_identity levels) = 23

Date levels = 3

Observations = 69

Conditional R^2^ = 0.603

Marginal R^2^ = 0.110

**Figure S6. DHARMa diagnostic plots for Model 5A.** The plots assess residual uniformity (Q-Q), residuals versus predicted values, dispersion, and outliers. Overall diagnostics indicated no major violations.

**Model 5B:** Active-minute experiment: Captive adult sand knifefish active minutes per night predicted by residual body condition on the final (5^th^) full moon

Generalised linear mixed-effect model (GLMM) fitted using package *glmmTMB* (R syntax):

Distribution family: negative binomial (nbinom2; *glmmTMB* terminology), log link

*active_minute ~ residual_condition + (1|fish_identity) + (1|date)*

Variables:

- active_minute (response) = number of active minutes within the observation night (count)
- residual_condition (predictor) = the residual body condition measurement most recent to the test date (continuous)
- fish_identity (random effect) = individual identification number (categorical)
- date (random effect) = date of the measurement (categorical) (see the Model 5A section above for the rationale)

**Table S6. Model 5B output.** Significant *P*-values in bold.

| **Effect type** | **Term** | **Estimate** | **SE** | ***z*-value** | **SD** | ***P*-value** |
| --- | --- | --- | --- | --- | --- | --- |
| Fixed | *(Intercept)* | 2.220 | 0.688 | 3.225 | NA | **0.0013** |
| Fixed | *residual_condition* | -10.294 | 2.867 | -3.590 | NA | **0.00033** |
| Random | *fish_identity* | — | — | — | 0.374 | — |
| Random | *date* | — | — | — | 0.671 | — |

N (fish_identity levels) = 23

Date levels = 3

Observations = 69

Conditional R^2^ = 0.553

Marginal R^2^ = 0.272

**Figure S7. DHARMa diagnostic plots for Model 5B.** The plots assess residual uniformity (Q–Q), residuals versus predicted values, dispersion, and outliers. A mild deviation in residuals versus predicted values was detected by the combined adjusted quantile test, whereas dispersion and outlier tests were non-significant.

**Model 5C:** Active-minute experiment: Captive adult sand knifefish active minutes per night predicted by residual body condition on the final (5^th^) new moon

Generalised linear mixed-effects model (GLMM) fitted using package *glmmTMB* (R syntax):

Distribution family: Gaussian, identity link

*active_minute ~ residual_condition + (1|fish_identity)*

Variables:

- active_minute (response) = number of active minutes within the observation night (count)
- residual_condition (predictor) = the residual body condition measurement most recent to the test date (continuous)
- fish_identity (random effect) = individual identification number (categorical)

Note:

The number of active minutes is formally a count response. A Gaussian family with an identity link was used because active-minute values were large and approximately continuous and this model fitted better than Poisson and negative binomial alternatives. The Gaussian model nevertheless showed a mild deviation in residuals versus predicted values (figure S8); the count models showed more systematic lack of fit.

**Table S7. Model 5C output.** Significant *P*-values in bold.

| **Effect type** | **Term** | **Estimate** | **SE** | ***z*-value** | **SD** | ***P*-value** |
| --- | --- | --- | --- | --- | --- | --- |
| Fixed | *(Intercept)* | 103.43 | 13.43 | 7.699 | NA | **1.37E-14** |
| Fixed | *residual_condition* | 316.58 | 195.54 | 1.619 | NA | 0.105 |
| Random | *fish_identity* | — | — | — | 55.41 | — |

N (fish_identity levels) = 23

Observations = 69

Conditional R^2^ = 0.825

Marginal R^2^ = 0.092

**Figure S8. DHARMa diagnostic plots for Model 5C.** The plots assess residual uniformity (Q–Q), residuals versus predicted values, dispersion, and outliers. A mild but significant deviation in residuals versus predicted values was detected by the combined adjusted quantile test, whereas dispersion and outlier tests were non-significant.

**Model 5D (Exploratory model):** Active-minute experiment: Captive adult sand knifefish active minutes per night predicted by residual body condition, sex, and their interaction, on the final (5^th^) full moon

Generalised linear mixed-effects model (GLMM) fitted using package *glmmTMB* (R syntax):

Distribution family: negative binomial (nbinom1; *glmmTMB* terminology), log link

*active_minute ~ residual_condition * sex + (1|fish_identity) + (1|date)*

Variables:

- active_minute (response) = number of active minutes within the observation night (count)
- residual_condition (predictor) = residual body condition measurement most recent to the test date (continuous)
- sex (predictor) = male or female (categorical)
- fish_identity (random effect) = individual identification number (categorical)
- date (random effect) = date of the measurement (categorical) (see the Model 5A section above for the rationale)

Note: The exploratory sex analysis was restricted to the RBC range represented in both sexes. The three lowest-RBC adults were all female and lay outside the RBC range represented by males, so they were excluded. The remaining 20 individuals included males and females spanning an approximately common RBC range (−0.1 to 0.1), allowing comparison between sexes over comparable condition values.

**Table S8. Model 5D output.** Significant *P*-values in bold.

| **Effect type** | **Term** | **Estimate** | **SE** | ***z*-value** | **SD** | ***P*-value** |
| --- | --- | --- | --- | --- | --- | --- |
| Fixed | *(Intercept, female level)* | 2.562 | 0.504 | 5.089 | NA | **3.5E-7** |
| Fixed | *residual_condition* | -5.626 | 3.057 | -1.840 | NA | 0.0657 |
| Fixed | *sex (male level)* | -0.481 | 0.283 | -1.703 | NA | 0.089 |
| Fixed | *residual_condition: sex (male level)* | 6.064 | 4.155 | 1.459 | NA | 0.145 |
| Random | *fish_identity* | — | — | — | 4.2E-5 | — |
| Random | *date* | — | — | — | 0.763 | — |

N (fish_identity levels) = 20

Date levels = 3

Observations = 60

Conditional R^2^ = not estimable

Marginal R^2^ = 0.157

**Figure S9. DHARMa diagnostic plots for Model 5D.** The plots assess residual uniformity (Q–Q), residuals versus predicted values, dispersion, and outliers. Overall, diagnostics indicated no major violations.

**Model 6A:** Active-minute experiment: Captive juvenile sand knifefish active minutes per night predicted by residual body condition, on the final (2^nd^) full moon

Generalised linear mixed-effects model (GLMM) fitted using package *glmmTMB* (R syntax):

Distribution family: Poisson, log link

*active_minute ~ residual_condition + (1|fish_identity) + (1|date)*

Variables:

- active_minute (response) = number of active minutes during nighttime (count)
- residual_condition (predictor) = the residual body condition measurement most recent to the test date (continuous)
- fish_identity (random effect) = individual identification number (categorical)
- date (random effect) = date of the measurement (categorical) (see the Model 5A section above for the rationale)

**Table S9. Model 6A output.** Significant *P*-values in bold.

| **Effect type** | **Term** | **Estimate** | **SE** | ***z*-value** | **SD** | ***P*-value** |
| --- | --- | --- | --- | --- | --- | --- |
| Fixed | *(Intercept)* | 4.157 | 0.183 | 22.771 | NA | **<2E-16** |
| Fixed | *residual_condition* | -0.585 | 1.544 | -0.379 | NA | 0.705 |
| Random | *fish_identity* | — | — | — | 0.696 | — |
| Random | *date* | — | — | — | 0.098 | — |

N (fish_identity levels) = 21

Date levels = 3

Observations = 63

Conditional R^2^ = 0.971

Marginal R^2^ = 0.007

**Figure S10. DHARMa diagnostic plots for Model 6A.** The plots assess residual uniformity (Q–Q), residuals versus predicted values, dispersion, and outliers. Overall, diagnostics indicated no major violations.

**Model 6B**: Active-minute experiment: Captive juvenile sand knifefish active minutes per night predicted by residual body condition, on the final (2^nd^) new moon

Generalised linear mixed-effects model (GLMM) fitted using package *glmmTMB* (R syntax):

Distribution family: Gaussian, identity link

*active_minute ~ residual_condition + (1|fish_identity)*

Variables:

- active_minute (response) = number of active minutes during nighttime (count)
- residual_condition (predictor) = the residual body condition measurement most recent to the test date (continuous)
- fish_identity (random effect) = individual identification number (categorical)

Note:

The number of active minutes is formally a count response. A Gaussian family with an identity link was used because active-minute values were large and approximately continuous and this model fitted better than Poisson and negative binomial alternatives. The Gaussian model nevertheless showed mild deviations in residual uniformity and residuals versus predicted values (figure S11); the count models showed more systematic lack of fit.

**Table S10. Model 6B output.** Significant *P*-values in bold.

| **Effect type** | **Term** | **Estimate** | **SE** | ***z*-value** | **SD** | ***P*-value** |
| --- | --- | --- | --- | --- | --- | --- |
| Fixed | *(Intercept)* | 511.71 | 36.06 | 14.190 | NA | **<2E-16** |
| Fixed | *residual_condition* | -555.31 | 396.92 | -1.399 | NA | 0.162 |
| Random | *fish_identity* | — | — | — | 123.68 | — |

N (fish_identity levels) = 21

Observations = 63

Conditional R^2^ = 0.761

Marginal R^2^ = 0.073

**Figure S11. DHARMa diagnostic plots for Model 6B.** The plots assess residual uniformity (Q–Q), residuals versus predicted values, dispersion, and outliers. Mild deviations in residual uniformity (KS test) and residuals versus predicted values (combined adjusted quantile test) were detected, whereas dispersion and outlier tests were non-significant.

**Model 7A:** Active-minute experiment: Captive sand knifefish active minutes per night predicted by residual body condition, life stage, and their interaction on the final full moon (5th for adults and 2nd for juveniles).

Generalised linear mixed-effects model (GLMM) fitted using package *glmmTMB* (R syntax):

Distribution family: negative binomial (nbinom1; *glmmTMB* terminology), log link

*active_minute ~ residual_condition * life_stage + (1|fish_identity) + (1|date)*

Variables:

- active_minute (response) = number of active minutes within the observation night (count)
- residual_condition (predictor) = the residual body condition measurement most recent to the test date (continuous)
- life_stage (predictor) = category of either adult or juvenile
- fish_identity (random effect) = individual identification number (categorical)
- date (random effect) = date of the measurement (categorical) (see the Model 5A section above for the rationale)

**Table S11. Model 7A output.** Significant *P*-values in bold.

| **Effect type** | **Term** | **Estimate** | **SE** | ***z*-value** | **SD** | ***P*-value** |
| --- | --- | --- | --- | --- | --- | --- |
| Fixed | *(Intercept) (reference = juvenile)* | 4.168 | 0.250 | 16.951 | NA | **< 2e-16** |
| Fixed | *residual_condition* | -1.249 | 1.443 | -0.866 | NA | 0.387 |
| Fixed | *life_stage (adult)* | -1.643 | 0.373 | -4.405 | NA | **1.06e-05** |
| Fixed (interaction) | *residual_condition:life_stage* | -6.197 | 2.756 | -2.248 | NA | **0.0246** |
| Random | *fish_identity* | — | — | — | 0.550 | — |
| Random | *date* | — | — | — | 0.309 | — |

* Changing the reference level to adult (instead of juvenile), the *residual_condition* slope output was: Estimate = -7.446, SE = 2.351, z-value = -3.167, P-value = 0.00154

N (fish_identity levels) = 44

Date levels = 6

Observations = 132

Conditional R^2^ = 0.954

Marginal R^2^ = 0.814

**Figure S12. DHARMa diagnostic plots for Model 7A.** The plots assess residual uniformity (Q–Q), residuals versus predicted values, within-group uniformity and variation, dispersion, and outliers. Overall, diagnostics indicated no major violations.

**Model 7B:** Active-minute experiment: Captive sand knifefish active minutes per night predicted by residual body condition, life stage, and their interaction on the final new moon (5th for adults and 2nd for juveniles).

Generalised linear mixed-effects model (GLMM) fitted using package *glmmTMB* (R syntax):

Distribution family: Gaussian identity link

*active_minute ~ residual_condition * life_stage + (1|fish_identity)*

Variables:

- active_minute (response) = number of active minutes within the observation night (count)
- residual_condition (predictor) = the residual body condition measurement most recent to the test date (continuous)
- life_stage (predictor) = category of either adult or juvenile
- fish_identity (random effect) = individual identification number (categorical)

**Table S12. Model 7B output.** Significant *P*-values in bold.

| **Effect type** | **Term** | **Estimate** | **SE** | ***z*-value** | **SD** | ***P*-value** |
| --- | --- | --- | --- | --- | --- | --- |
| Fixed | *(Intercept) (reference = juvenile)* | 511.71 | 27.43 | 18.652 | NA | **< 2e-16** |
| Fixed | *residual_condition* | -555.30 | 301.97 | -1.839 | NA | 0.0659 |
| Fixed | *life_stage (adult)* | -408.28 | 35.92 | -11.366 | NA | **< 2e-16** |
| Fixed (interaction) | *residual_condition:life_stage* | 871.89 | 452.92 | 1.925 | NA | 0.0542 |
| Random | *fish_identity* | — | — | — | 94.37 | — |

* Changing the reference level to adult (instead of juvenile), the *residual_condition* slope output was: Estimate = 316.58, SE = 337.56, z-value = 0.938, P-value = 0.3483

N (fish_identity levels) = 44

Observations = 132

Conditional R^2^ = 0.621

Marginal R^2^ = 0.380

**Figure S13. DHARMa diagnostic plots for Model 7B.** The plots assess residual uniformity (Q–Q), residuals versus predicted values, dispersion, and outliers. The combined adjusted quantile test detected residual structure. Tests of within-group uniformity and homogeneity of variance were significant, as was the outlier test; the overall dispersion test was non-significant.

**Model 8**: Light-ramping experiment: Captive adult sand knifefish moonlight illuminance at moment of hiding (sand burying) predicted by residual body condition

Logistic nonlinear mixed-effects model (NLMER) fitted using package *lme4* (R syntax):

*SSfpl(condition, A, B, xmid, scal) ~ (xmid|fish_identity) + (A|fish_identity)*

*SSfpl* is a self-starting four-parameter logistic function:

$$hiding\_illuminance \sim B+\frac{A-B}{1+exp\left( \frac{xmid-residual\_condition}{scal} \right)}$$

Variables:

- hiding_illuminance (response) = ground illuminance level (lx) during the 30 min light ramp that triggered hiding (continuous)
- residual_condition (predictor) = the residual body condition measurement most recent to the test date (continuous)

Random effect parameters:

To account for the repeated-measures design, fish identity (fish_identity) was treated as a random effect. In logistic NLMERs, random effects can in principle be applied to all four parameters (A, B, xmid, scal), but such a complex model did not converge in this setup. We were able to include fish identity as random effects for A (left asymptote) and xmid (inflection point). Allowing A to vary among fish was especially important because the hiding-threshold illuminance of low-condition individuals showed substantial between-trial variation.

Parameters:

- A = left asymptote (the upper response asymptote in this fitted decreasing curve)
- B = right asymptote (the lower response asymptote)
- xmid = inflection point position
- scal = scale parameter controlling transition steepness; for a fixed difference between asymptotes, smaller absolute values produce steeper transitions

**Table S13. Model 8 output.** 95% CIs obtained using the Wald method.

| **Effect type** | **Term** | **Estimate** | **SE** | **t-value** | **Lower 95% CI** | **Upper 95% CI** | **SD** |  |
| --- | --- | --- | --- | --- | --- | --- | --- | --- |
| Fixed | *A* | 0.0412 | 0.0034 | 12.28 | 0.0346 | 0.0477 | NA | |
| Fixed | *B* | 0.0029 | 0.0012 | 2.47 | 0.0006 | 0.0051 | NA | |
| Fixed | *xmid* | -0.0168 | 0.0018 | -9.25 | -0.0204 | -0.0133 | NA | |
| Fixed | *scal* | 0.0084 | 0.0016 | 5.21 | 0.0052 | 0.0115 | NA | |
| Random | *A\|fish_identity* | — | — | — |  |  | 0.0110 | |
| Random | *xmid\|fish_identity* | — | — | — |  |  | 9.383E-08 | |

N (fish_identity levels) = 21

Observations = 180

Marginal R^2^ = 0.762

**References**

1.	Poon L, Crampton WGR. 2026 Timescapes of fear: Exogenous and endogenous control of moonlight-mediated circalunar foraging rhythms in a weakly electric fish. *Biology Letters* 22(4), 20250704 .
